# Cellulose-Based Scattering Enhancers for Light Management Applications

**DOI:** 10.1021/acsnano.1c09198

**Published:** 2022-04-27

**Authors:** Han Yang, Gianni Jacucci, Lukas Schertel, Silvia Vignolini

**Affiliations:** Department of Chemistry, University of Cambridge, Lensfield Road, Cambridge CB2 1EW, United Kingdom

**Keywords:** whiteness, transparency, cellulose particles, scattering, optical haze, light transport

## Abstract

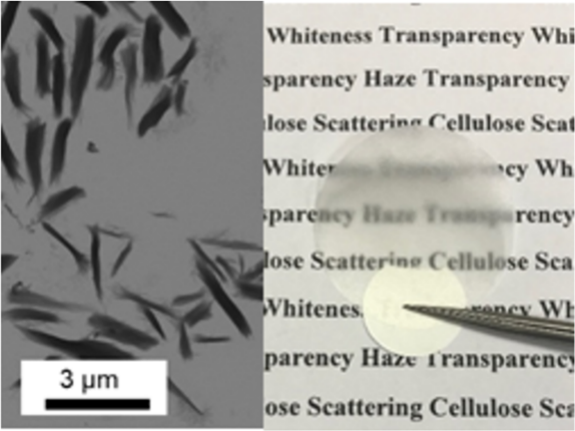

To manipulate the light–matter interaction effectively, we often rely on high refractive index inorganic nanoparticles. Such materials are contained essentially in everything that looks colorful or white: from paints to coatings but also in processed food, toothpaste, and cosmetic products. As these nanoparticles can accumulate in the human body and environment, there is a strong need to replace them with more biocompatible counterparts. In this work, we introduce various types of cellulose-based microparticles (CMPs) of four sizes with optimized dimensions for efficient light scattering that can replace traditional inorganic particles. We demonstrate that the produced materials can be exploited as highly efficient scattering enhancers, with designed optical performance. Finally, exploiting these cellulose colloids, we fabricated scattering materials and high transmittance/haze films with record performances with respect to the state-of-the-art values. The renewable and biocompatible nature of our systems, combined with their excellent optical properties, allows for the use of our cellulose-based particles in paints, LEDs, and solar cell devices and especially in applications where the biocompatibility of the component is essential, such as in food and pharmaceutical coatings.

The appearance of nonabsorbing materials, from transparency to whiteness or haze, can be designed by engineering the internal structure at the nano- and microscale. Most common, in industrial settings, is the use of light scattering particles assembled into macroscale structures. Controlling the size and morphology of these scattering enhancers to produce materials with different appearances from white to opaque or hazy is essential for many products in our daily life.^1^ Haze is the percentage of the ratio of the light passing through a material that has been diffusely scattered to the total transmitted light; haze can be measured and calculated as shown in Figure S1. Paints, paper, cosmetics, and the food industry are just some examples of where scattering enhancers are in wide use,^2−4^ while the ability to optimize haze has huge implications for optoelectronic (e.g., as light management layers for solar cells to enhance light absorption or for organic light-emitting diodes to evenly distribute light) and displays applications removing the glare effect by reducing specular reflection.^5,6^

So far, a huge variety of approaches and materials have been developed to increase the scattering properties of materials.^7^ However, the most common industrial approach, which is at the base of every white pigment, consists of using high refractive index titanium dioxide (TiO_2_) nanoparticles.^8^ However, these are under increased scrutiny in terms of their biocompatibility.^2,9^ TiO_2_ nanoparticles have been classified as a category 2 carcinogen by inhalation by the EU in 2020, and many research results also support this claim.^10^ Recently, several titanium-dioxide-free, highly scattering films have been developed either using polymeric materials,^11,12^ including also biopolymers, such as cellulose nanofibers,^13^ or cellulose derivatives.^14^ Similarly, cellulose nanofibers have also been exploited to produce high optical haze in materials with good transparency.^15,16^ Although cellulose has a low refractive index (the average refractive index of cellulose is about 1.56),^17^ by designing the morphology of the particles, we can optimize their scattering efficiency for the desired application; see as an example the design of anisotropy by Jacucci et al.^18^

In this work, we introduce a type of cellulose-based microparticles (CMPs). Unlike the two major types of cellulose nanomaterials, such as cellulose nanofibers and nanocrystals, CMPs have a larger lateral size and a smaller aspect ratio to optimize the interaction with the wavelength of the visible spectrum of light. Here, we demonstrate that, by tuning the size of such particles, scattering performances can be optimized on the single scatter level. Moreover, by additionally controlling the spatial arrangement of these particles in a disordered network, both highly scattering and optical haze materials can be produced, outperforming current materials. We foresee that this class of cellulose particles will find a wide variety of optical applications allowing researchers to solve the biocompatibility problem not only in paints, coatings, and technological applications such as photovoltaic devices but also for personal care products. In fact, cellulose is biocompatible,^19,20^ and nanoparticles made from cellulose have been demonstrated to be not cytotoxic.^21,22^ Additionally, as the CMPs are much larger than regular cellulose nanofibers and nanocrystals, they can directly be compared to microcrystalline cellulose, which has been approved for many applications in the food and pharma sectors.^23^ Moreover, in contrast to conventional cellulose nanocrystals, CMPs can be obtained with milder hydrolysis conditions, reducing the acid consumption and the heat required.

## Results and Discussion

**Figure 1:**
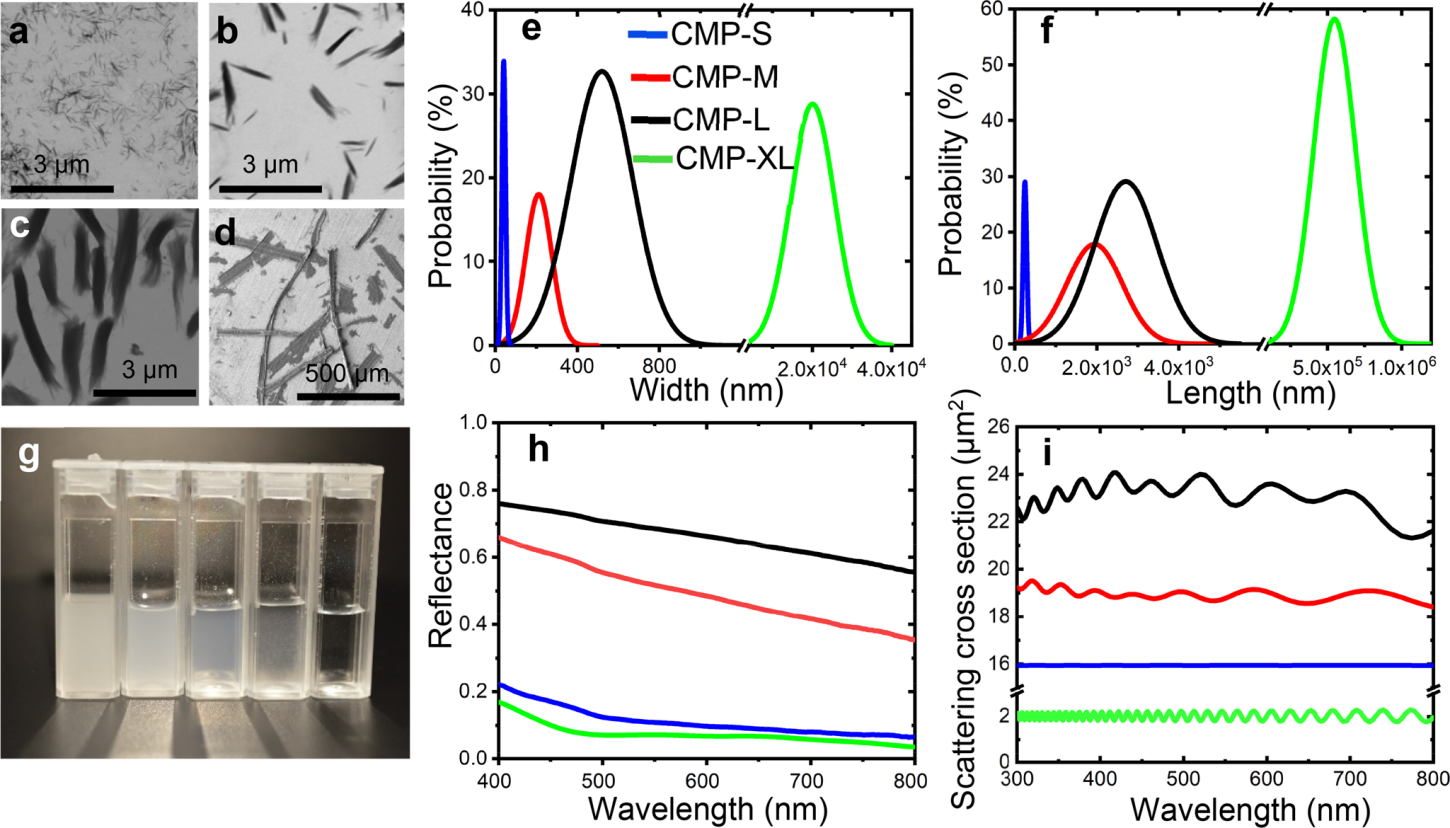
STEM images of (a) CMPs-S, (b) CMPs-M, (c) CMPs-L, and (d) CMPs-XL. Particle size distribution: (e) width distribution probability and (f) length distribution probability of CMPs-S, CMPs-M, CMPs-L, and CMP-XL, respectively (width and length were measured from STEM images). (g) Picture of light passing through suspensions of CMPs-L, CMPs-M, CMPs-S, CMPs-XL (concentration is 0.1% in weight percentage), and water (from left to right, illumination from the front). (h) Reflectance of the four cellulose CMP suspensions shown in the picture in part g, measured with an integrating sphere. (i) Optical simulations of the scattering cross section of the four different CMP building blocks.

We fabricated various cellulose-based microparticles of different sizes; the width and length distributions of CMPs are reported in Figure 1e and f and summarized in Table 1 (their histograms and fitted log–normal distribution curves are shown in Figure S2). The sizes of the different particles were designed in order to be used in two main applications: (1) highly scattering and (2) high haze, high transparency films. In general, light is best scattered by a single scattering Mie sphere, so a low aspect ratio (ideally 1) would be best for achieving high scattering strength at the single scatterer level. However, it is not ideal to have spherical scatterers to build solid stable networks for making highly scattering films. Thus, small aspect ratios of anisotropic particles are the ideal scatterers for the mentioned applications. We, therefore, used a starting set of targeted parameters identified in one of our previous numerical studies.^18^

**Table 1:** Dimensions of Various Cellulose Particles^a^

cellulose particles	width	length	thickness
CMPs-S	40 ± 9 nm	228 ± 32 nm	13 ± 2.3 nm
CMPs-M	212 ± 64 nm	1944 ± 677 nm	84 ± 26 nm
CMPs-L	520 ± 151 nm	2706 ± 767 nm	174 ± 58 nm
CMPs-XL	20 ± 5.5 μm	547 ± 140 μm	2.8 ± 0.6 μm

a The width and length were obtained with STEM, and the thickness was obtained with the cross section of SEM.

The three types of CMPs, namely CMPs-L (large width), CMPs-M (medium width), and CMPs-S (small width), were prepared by H_2_SO_4_ acid hydrolysis from both microcrystalline cellulose and cotton. Briefly, microcrystalline cellulose powder or cellulose filter paper (cotton) was hydrolyzed with sulfuric acid and then quenched by adding water, followed by a purification step. Sequential centrifugation was used for narrowing the particle size distribution. For details, see the Methods part of the Experimental Section.

In the case of haze/transparency, in contrast, a low aspect ratio for large particles can be beneficial, as each particle is transparent but their assembly scatters the light a bit without changing the propagation direction of light too much. The designed CMPs-XL are better than other nanocelluloses for haze applications, as their “bulk” avoids too much scattering. To achieve such even larger particles with micron-sized diameter, so-called CMPs-XL, we used TEMPO oxidation of cotton fibers. TEMPO oxidation is a one-step reaction that selectively converts the hydroxyl groups on C6 of the cellulose glucose ring into negatively charged carboxyl groups. As a result, this treatment increases the repulsion force and decreases the hydrogen bonding among the native cellulose nanofibers, resulting in fibers with a width of around 20 μm (SEM image in Figure 1d).

The three smaller types of CMPs show a low aspect ratio of 4–6, making them ideal candidates for use as scattering enhancers. Having such a small aspect ratio is important especially in suspension, where the radius of gyration determines the scattering “size”. Particles that are very long or with a very small diameter would have a decreased scattering efficiency, as in the case of other cellulose nanomaterials. In fact, it is important to note that the dimensions of the produced CMPs strongly differ from traditional cellulose nanocrystals (CNCs), which are 3–5 nm in width and 100–200 nm in length,^24^ or cellulose nanofibers (CNFs), which are generally 3–20 nm in width and a few micrometers in length.^25−27^

Such difference in the size results in different scattering abilities of the particles, making the fabricated CMPs ideal scattering enhancers. The efficiency of the produced CMPs as a single scatterer can be observed in Figure 1g, where suspensions of CMPs of different sizes are dispersed in water with a fixed concentration (0.1% in weight percentage). The whiteness observed in the macroscopic image of the particle suspensions is a direct indicator of the scattering ability of the single particles. Clearly, a gradient from CMPs-L to CMPs-M and CMPs-S to CMPs-XL can be observed. This observation can be quantitatively assessed in the optical measurements of reflectance (see Figure 1h). We find that, up to a width of ∼500 nm, larger particles have a larger scattering cross section,^28^ while increasing further the size of CMPs decreases their scattering strength (green curve in Figure 1h). According to the Mie theory for scatterers^28^ (Figure S3), the reflectance of CMPs becomes nonmonotonic with particle size, which is consistent with our result shown in Figure 1h.

To further confirm that the scattering properties of CMPs are optimal, we performed optical simulations of the scattering cross sections in Figure 1i of flake-shaped anisotropic particles of the same size as extracted from Figure 1e and f (the scattering cross section is defined as the total scattered power divided by the power per unit area of the incident beam). Despite the fact that the fabricated CMPs are thinner in the third dimension, they can be approximated as cylinders for the optical simulation. CMPs-L show an angular distribution of the scattered light, which is asymmetric (Mie scattering), while in contrast for CMPs-S and CMPs-M the scattering is symmetric (cf. Figure S4), resembling Rayleigh scatterers, explaining their difference in scattering strength. Additionally, for comparison we report the value of Mie scattering for traditional cellulose nanocrystals in Figure S5; as we can see, the CNCs have a much smaller cross section (around 0.3 μm^2^) compared to the CMPs (around 23 μm^2^ for CMPs-L) while preserving the Rayleigh scattering behavior in terms of angular cross section.

**Figure 2:**
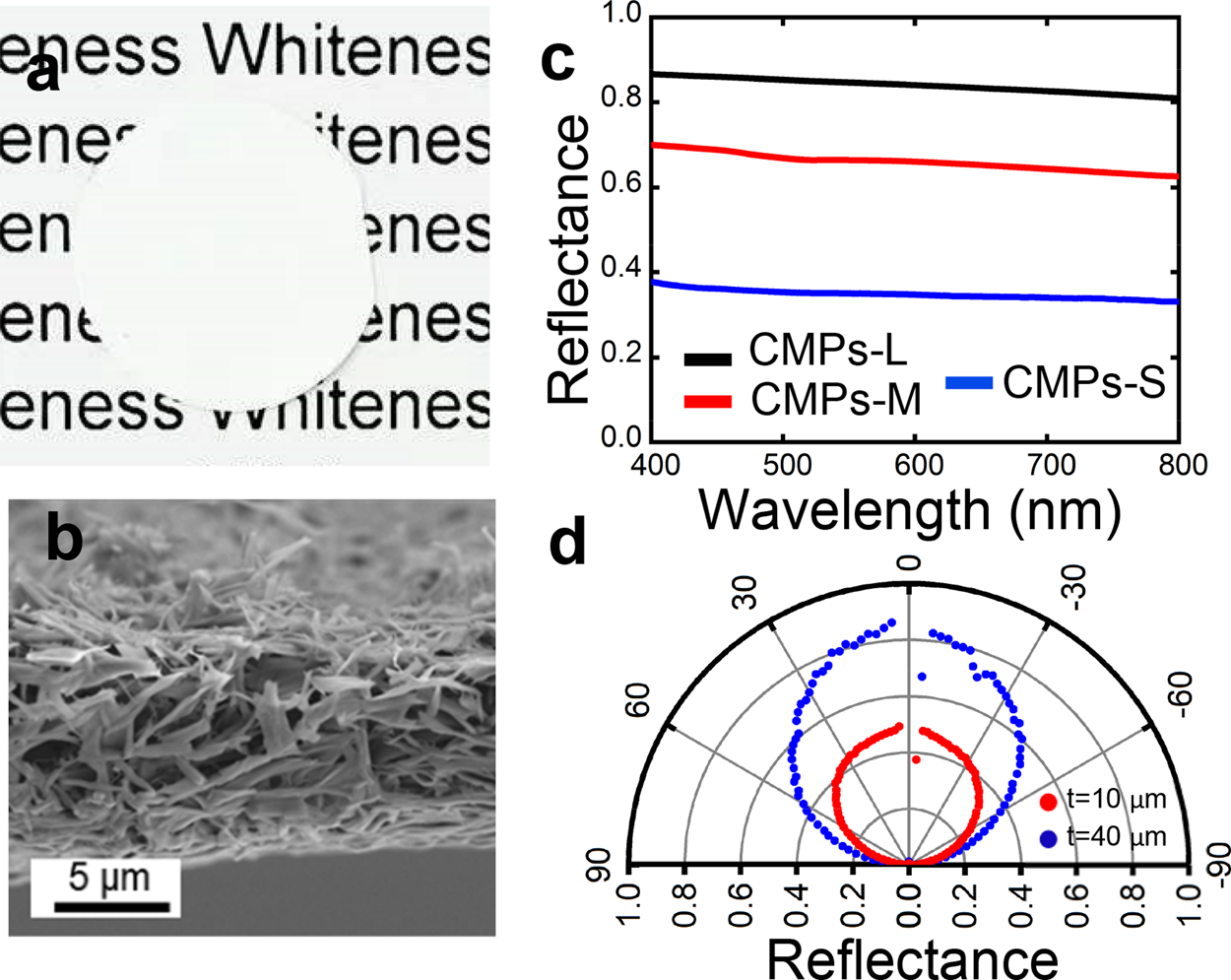
(a) Picture of a typical white film (9 μm in thickness) made from CMPs-L particles. The text underneath this film is hard to be resolved even when the center part of the film is closely touched with the background paper. (b) SEM image of the cross section of the white film shown in part a. (c) Reflectance of white films made of CMPs-L, CMPs-M, and CMPs-S at the same thickness of 25 μm and ff = 25%, measured with an integrating sphere. (d) Angular distribution of the intensity (wavelength = 400 nm) reflected by films made of CMPs-L particles by goniometer measurement. Even for the lowest thickness, CMPs-L-based materials follow a Lambertian distribution. Intensity normalized to a white diffuser.

To showcase the optical performances and the exceptional scattering strength of CMPs, we also produced highly scattering porous thin films (Figure 2a and b). It is well-known that for a fixed thickness, the scattering efficiency of a white material is determined by both its filling fraction and the size of its building blocks.^18^ Therefore, we first tested the different sized CMPs (S, M, and L) to produce free-standing films using vacuum filtration followed by freeze-drying, as shown in Figure S6. Figure 2c shows the optical response of films made with CMPs-L, M, and S with fixed values of the thickness and the filling fraction of 25 μm and 25%, respectively. Once we identified that the CMPs-L provided the best whiteness in thin films, the same particles were turned into partially hydrophobic material by modification with trichloromethylsilane vapor and porous films were achieved by simply drop-casting them on a substrate once suspended in ethanol; see the extended discussion in the Supporting Information and Figure S7.

The scattering efficiency of the CMP-based films and the derivation of the transport mean free path are discussed in the Supporting Information and Figure S8. As expected, the reflectance is maximized for larger particles, following the scattering behavior of the single particles (Figure 1). In particular, CMPs-L films show a reflectance of around 85%, which is significantly larger than those of CMPs-M and CMPs-S, which can reflect only 70% and 40%, respectively. The scattering efficiency of the CMP-based films, in terms of their transport mean free path, is summarized in Table 2. This parameter represents the average distance that light has to travel in a medium before its initial propagation direction is randomized and is inversely proportional to the scattering efficiency.^29^ Therefore, the transport mean free path is a good measure of the scattering responses of different materials independent of the sample structural parameters. Table 2 shows that the obtained CMPs-L films exhibit a value of transport mean free path as low as 1 μm, which is the smallest value of transport mean free path ever reported for low refractive index scattering media.^11,30^ It is important to also highlight that the latter processes are (i) less energy consuming and (ii) more scalable as they allow researchers to skip the solvent exchange methods that have been previously developed to produce porous scattering cellulosic materials.^13^ Moreover, the value of the transport mean free path is comparable with what is reported for spherical TiO_2_ particles in air,^31^ despite the fact that the latter might be further optimized by introducing anisotropy.

**Table 2:** Transport Mean Free Path and Whiteness Values for Different Cellulose-Based Materials^a^

cellulose particles	*ff*	*l*_*t*_ (μm)	*t* (μm)	*W*
CMPs-S	0.24 ± 0.01	21.4 ± 1.0	25.8 ± 1.2	66
CMPs-M	0.25 ± 0.01	6.8 ± 0.3	24.5 ± 1.3	83
CMPs-L	0.25 ± 0.01	2.6 ± 0.04	24.8 ± 0.5	89
CMPs-L	0.39 ± 0.02	1.6 ± 0.1	9.2 ± 0.4	84
CMPs-L	0.53 ± 0.02	0.99 ± 0.05	9.0 ± 0.5	88
CNFs			9	86

a The thickness was obtained from SEM, the filling fraction was calculated by the method described in the Characterizations section, and the transport mean free path was obtained by the method described in the Characterizations section. The whiteness of CNF systems was calculated from the raw data in ref (13), where *ff*, *l*_*t*_, *t*, and *W* are the filling fraction, mean free path, thickness, and whiteness, respectively.

The filling fraction and thickness of the films were controlled by the initial amount of CMPs-L and the duration of the vacuum process. The SEM images of Figure 2b and Figure S9a show films with a comparable thickness (9 μm) and different filling fractions, *ff* = 40% and *ff* = 53%, respectively. As depicted in Figure S9e and predicted by the numerical results in Figure S10a, for CMPs-L, increasing the filling fraction leads to an increase of reflectance from around 71% to 77% at 600 nm. In such CMPs-L films, the random 3D network is due to the hydrogen bonding forming between CMPs-L, and the micropores are the results of the formation of ice crystals during the freezing step.^32^

Finally, the optical response of the produced CMPs-L films was evaluated in terms of angular dependence. The angular distribution of reflected light was determined using a goniometer setup (see Methods). The illumination angle was fixed at normal incidence, and the angular distribution of the intensity was acquired by rotating the detector arm around the sample. Figure 2d shows that the produced films follow a Lambertian profile of the ideal diffuser, even for a very thin film.

**Figure 3:**
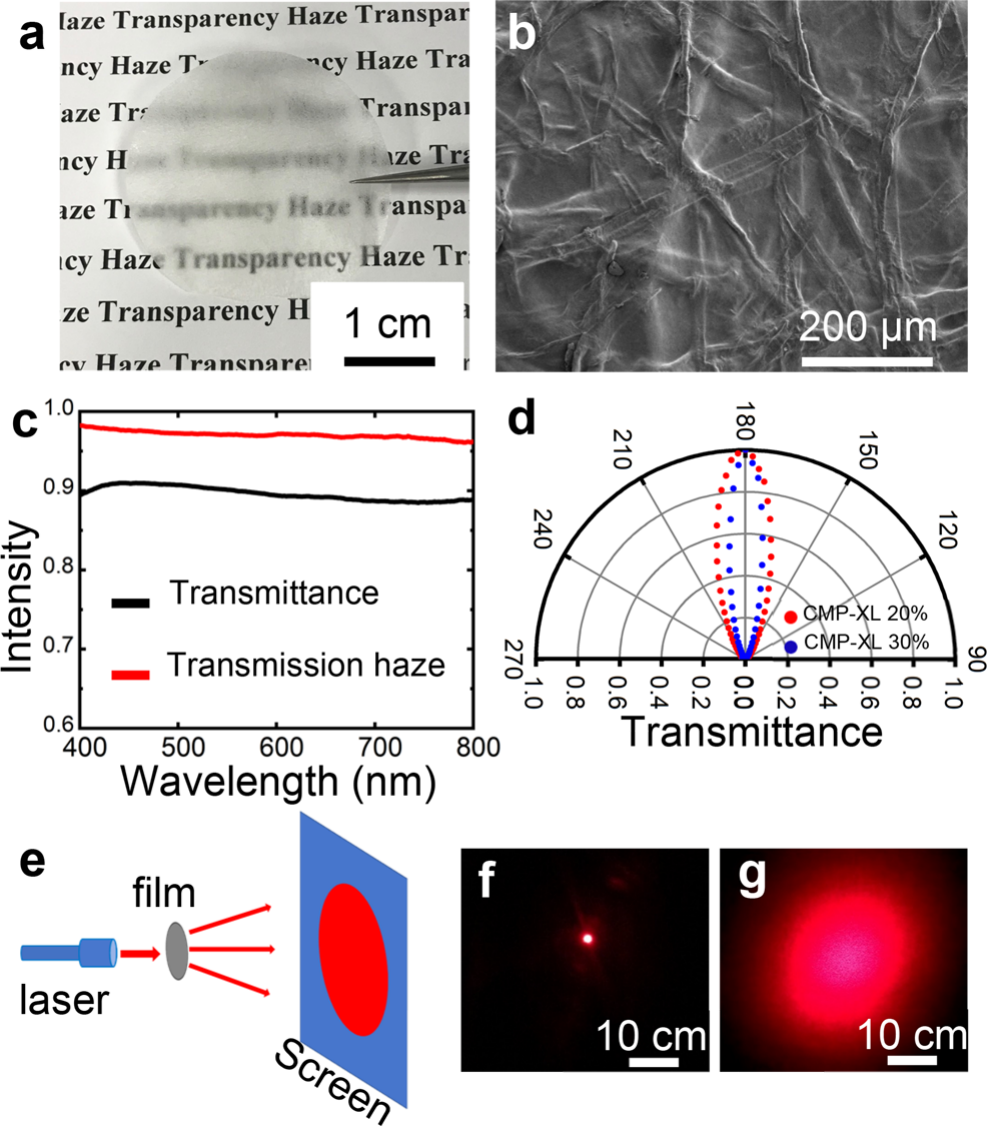
(a) Photograph of a high optical haze film (with 20% CMPs-XL). The upside part of the film is about 1 cm above the background. (b) SEM image of the surface of a composite film with 20% CMPs-XL. (c) Transmittance and haze spectra of a composite film with 20% CMPs-XL, measured with an integrating sphere. (d) Angular distribution of the intensity (wavelength = 400 nm) transmitted through films made of CMPs-XL by goniometer measurement. Both 20% CMPs-XL and 30% CMPs-XL samples show transmitted light outside the ballistic direction (180°). Intensity normalized to the maximum of the curve. (e) Schematic of the setup for showing the light scattering effect of the prepared films. The light scattering effect of (f) a pure CMC film and (g) a composite film with 20% CMPs-XL when a laser with a diameter of 0.2 cm passes through.

While increasing further the size of CMPs decreases their scattering strength (Figure 1h), we observed that CMPs-XL, with micron-size morphology, are good candidates for high haze, as their forward scattering is enhanced. Therefore, we prepared films with high transmittance and ultrahigh haze by mixing CMPs-XL into the carboxymethyl cellulose matrix. A typical composite film with 20% CMPs-XL doping (50 μm in thickness) is shown in Figure 3a. While the bottom of the film, which is in contact with the background, shows high transparency; the upper part, which is away from the background, reveals the haze effect (as the text appears blurry). The optical performance of the films is shown in Figure 3c. In contrast, a pure carboxymethyl cellulose film is fully transparent; see Figure S11. As shown in the SEM image in Figure 3b, CMPs-XL were uniformly distributed in the CMC matrix, serving as scatting elements to improve the optical haze. The shape of the scattering cross section of CMPs-XL and the small refractive index contrast between scatterers and matrix simultaneously guarantee high transparency and very high haze.

The angular distribution of light transmitted through a typical film made of CMPs-XL reported in Figure 3d shows that the illumination beam is also transmitted at nonballistic angles. Figure 3g shows how collimated light from a laser beam (diameter of 0.2 cm) is diffused by CMP-XL films, forming a homogeneously illuminated circular area with a diameter of over 30 cm at a distance of 40 cm from the film (see Figure 3e for the setup). In contrast, in the absence of CMPs-XL, the light is only slightly scattered (Figure 3f).

Moreover, the transmittance and haze can be easily adjusted by changing the weight ratio of CMPs, as shown in Figure S12. The optical haze of different films can vary from 33% to 98%, while maintaining the transmittance around 90%. The optical properties of various transparent and haze cellulose films are summarized in Table S1. It is important to notice that, when compared with other reported systems, our films reach a transmittance of 89–92% at 400–800 nm and an optical haze of 96–98% at 400–800 nm, which are the highest values reported so far in the literature.

## Conclusion

In conclusion, we produced a type of cellulose material, cellulose micron particles. By tailoring their size, these particles can be implemented to engineer light transport and produce highly reflective white materials to fully transparent films with high optical haze. The single particle scattering performances have been experimentally optimized in agreement with the results of the optical simulations. Additionally, assemblies of CMPs were able to achieve materials with a scattering mean free path as small as ∼1 μm and high transmittance (92%) and haze (98%), outperforming the results previously reported in the literature. Therefore, we believe that these cellulose-based optical materials combined with the simplicity of the production can find applications in the next-generation sustainable, biocompatible, and renewable coatings, such as pigments in inks, for light distribution and harvesting devices and antiglaring materials.

## Experimental Section

### Materials

Microcrystalline cellulose (MCC) was purchased from SERVA Electrophoresis. Whatman No. 1 cellulose filter paper and sulfuric acid (concentration > 95%) were purchased from Fisher Chemical. Trichloromethylsilane (TCMS) was purchased from Sigma-Aldrich. Ethanol (absolute) was from VWR chemicals. Carboxymethyl cellulose (CMC, MW ∼ 90000) was from Acros. Polyvinylidene fluoride (PVDF) membrane (average pore size 0.45 μm) was purchased from Merck Millipore Ltd.

### Methods

#### Preparation of Cellulose Nanoparticles with Various Dimensions

Cellulose microparticles (CMPs-L) were prepared by acid hydrolysis. Briefly, cellulose microcrystalline powder (1 g) was hydrolyzed with sulfuric acid (50%, 60 mL) for 5 h at 50 °C and then quenched by adding 300 mL of milli-Q water. The acid supernatant was removed by centrifugation. The hydrolyzed cellulose particles were dispersed by adding 100 mL of milli-Q water and then centrifuged. This process was repeated three times to remove most of the acid, and the suspension of hydrolyzed cellulose particles was dialyzed against milli-Q water (MWCO 12–14 kDa) for 1 week with changing water two times a day. The suspension (0.5 wt %, 30 mL) was tip sonicated in an ice bath (Fisher brand ultrasonic disintegrator 500 W, amplitude 30%, 2 s on and 2 s off). The suspension was centrifuged at 2000 rpm for 5 min, and then the supernatant was collected and centrifuged at 3000 rpm for 5 min to obtain the cellulose nanoparticles with the required dimension. The cellulose nanoparticles with other dimensions were obtained by adjusting the concentration of sulfuric acid, reaction time, and temperature. The CMPs-M were obtained from the hydrolysis of MCC with 55% H_2_SO_4_ at 60 °C for 5 h. The CMPs-S were prepared from the hydrolysis of cellulose filter paper (Whatman No. 1) with 55% H_2_SO_4_ at 50 °C for half an hour.

#### Preparation of TEMPO-Oxidized Cellulose Fibers (CMPs-XL)

Whatman No. 1 cellulose filter paper was first ground into small pieces by a coffee grinder, followed by TEMPO oxidation. Briefly, 1 g of cellulose was suspended in 150 mL of milli-Q water, and 0.123 g of TEMPO, 1.23 g of NaBr, and 1.23 g of NaClO were added and stirred for 4.5 h at room temperature while the pH was kept at 10 by the addition of 1 M NaOH solution. The reaction was stopped by adjusting the pH to 6 with 5 M HCl, and then the oxidized cellulose fibers were washed by filtration and dialyzed against milli-Q water.

#### Fabrication of White Films

The white films were fabricated by vacuum filtration on a hydrophilic polyvinylidene fluoride (PVDF) membrane. Briefly, a given amount of dispersion of cellulose nanoparticles (0.5 mg/mL) was vacuum filtrated until a wet film with no visible water layer was formed and then was continuously vacuumed for a certain time. The filter membrane with the attached wet film was carefully taken off and transferred into liquid nitrogen. Finally, the frozen film was freeze-dried (Scanvac, Coolsafe) to yield a free-standing film.

#### Fabrication of Films with High Transmittance and Haze

The TEMPO-oxidized cellulose fibers were mixed with 1% CMC solution at various weight ratios. The mixture was first degassed in a vacuum chamber and then was cast on a Petri dish to obtain a free-standing film.

#### TCMS Vapor Treatment

The CMPs were converted into hydrophobic surfaces by treating them with TCMS vapor. In short, freeze-dried CMPs were put in the upper space of a chamber with 1 mL of TCMS liquid for 30 s. After TCMS treatment, CMPs were dispersed in ethanol by sonication, and then films were formed by casting this suspension in the air.

### Characterizations

#### Measuring the Size Distribution of CMPs by STEM

The size distribution of cellulose nanoparticles was measured by scanning transmission electron microscopy (STEM). A dilute suspension of CMPs (0.001%) was dropped on a carbon coated copper grid (300 mesh) for 2 min and removed by a piece of filter paper; then a drop of uranyl acetate solution (2%) was applied as stain for 1 min before being removed by a piece of filter paper. The samples were measured on a Mira3 system (TESCAN) operated at 30 kV and a working distance of 5 mm. The length and width of the nanoparticles were analyzed by ImageJ.

#### Estimating the Microstructure of White Films by SEM

The cross section of each film was measured by scanning electron microscopy (SEM) with a Mira3 system (TESCAN) operated at 5 kV and a working distance of about 6 mm. To prepare specimens, the films were frozen in liquid nitrogen and then cracked. The samples were mounted on aluminum stubs using conductive carbon tape and coated with a layer of platinum (10 nm in thickness) by a sputter coater (Quorum Q150T ES). The thickness of each film was determined from SEM images of their cross sections.

#### Integrating Sphere for Transmittance/Reflectance

The total transmittance measurements were performed with an integrating sphere (Labsphere). A light source (Ocean Optics HPX-2000) was coupled into an optical fiber (600 μm Thorlabs FC-UV100-2-SR) via a collimator (Thorlabs), and the signal was collected by a spectrometer (Avantes HS2048), as shown in Figure S1 (T_1_ and T_2_). The signal was normalized with respect to the intensity when no sample was mounted. The background was recorded when no light was applied. The range of wavelengths was between 400 and 800 nm. Five spectra were taken for each sample and averaged to reduce the signal-to-noise ratio. Each spectrum was recorded using an integration time equal to 3 s.

#### Haze Measurements by Integrating Sphere

Haze was measured using the same setup as for the total transmittance measurement, except that a port at 180° to the sample is opened when measuring the scattered light with and without sample, as shown in Figure S1.

#### Calculation of Filling Fraction

The filling fraction (*ff*) was calculated using a nominal density ρ of 1.5 g/cm^–3^ for cellulose; the volume of cellulose nanoparticles *v*_1_ = *m*/ρ (*m* is the weight of films). The volume of films *v*_2_ = π*r*^2^*d* was estimated by using the average thickness of the films *d*, and *r* is the radius of the films. The filling fraction is calculated by *ff* = *v*_1_/*v*_2_.

#### Numerical Simulation of the Optical Properties

2D structures with different types of disorder were generated using a recently developed inverse design algorithm discussed in detail by Jacucci et al.^11^ Numerical simulations of the optical response of the generated structures were then performed in Lumerical, a piece of software using the finite difference time domain (FDTD) method.

#### Transport Mean Free Path Measurements

The transport mean free path was evaluated from the total transmission data by means of the following equation:^11^
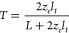
where *T*, *L*, *l*_*t*_, and *z*_*e*_ are the total transmission, thickness, mean free path, and extrapolation length, respectively. This latter parameter takes into account internal reflections at the sample’s interfaces on the evaluation of the mean free path and can be calculated by knowing the filling fraction system.^18,29,30,33^

#### Angular Distribution Measurements

The angular distribution of reflected/transmitted light shown in Figures 2d and 3d was determined using a goniometer. In particular, a xenon lamp (Ocean Optics HPX-2000) was coupled to an optical fiber (Thorlabs FC-UV100-2-SR) and shone onto the sample. The illumination angle was fixed at normal incidence, and the angular distribution of intensity was acquired by rotating the detector arm around the sample with a resolution of 1°. To detect the signal, a 600 μm core fiber (Thorlabs FC-UV600-2-SR) connected to a spectrometer (Avantes HS2048) was used. The spectra were averaged over 10 acquisitions to reduce the signal-to-noise ratio.

#### Contact Angle Test

The contact angle (CA, θ) was measured by using a drop shape analysis instrument (First Ten Angstroms, USA) at ambient temperature. A water droplet of 5 μL was placed on the surface of a sample, and the contact angle was an average of six measurements on different positions on the surface.

## Data Availability Statement

Additional data relating to this publication are available from the University of Cambridge data repository (https://doi.org/10.17863/CAM.83099).

## Supporting Information Available

Detailed schematic of the experimental setup for haze measurements; histograms and fitted log–normal distribution curves for the width and length of four types of CMPs; numerical, three-dimensional simulations of the single particle properties of CMPs; schematic of the process for hydrophobic treatment of CMPs-L, and reflectance and SEM images of the hydrophobic CMPs-L films; morphological characterization of CMP films; spectral scattering properties of CMP films; total light transmittance and haze values of films with different contents of CMPs-XL; table of transmittance and haze values of various transparent cellulose films at 400–800 nm (PDF)
