# Supplementary material for: Cellulose-Based Scattering Enhancers for Light Management Applications
Source: ACS Nano. 2022 Apr 27;16(5):7373–9. doi: 10.1021/acsnano.1c09198 (PMC9134489; doi:10.1021/acsnano.1c09198)
Supplement: Supplementary file 1 — nn1c09198_si_001.pdf [file nn1c09198_si_001.pdf]

Supporting information

Cellulose-based Scattering Enhancers for Light Management Applications

Han Yang, Gianni Jacucci, Lukas Schertel, Silvia Vignolini *

Department of Chemistry, University of Cambridge, Lensfield Road, Cambridge CB2 1EW, United Kingdom

*e-mail: sv319@cam.ac.uk

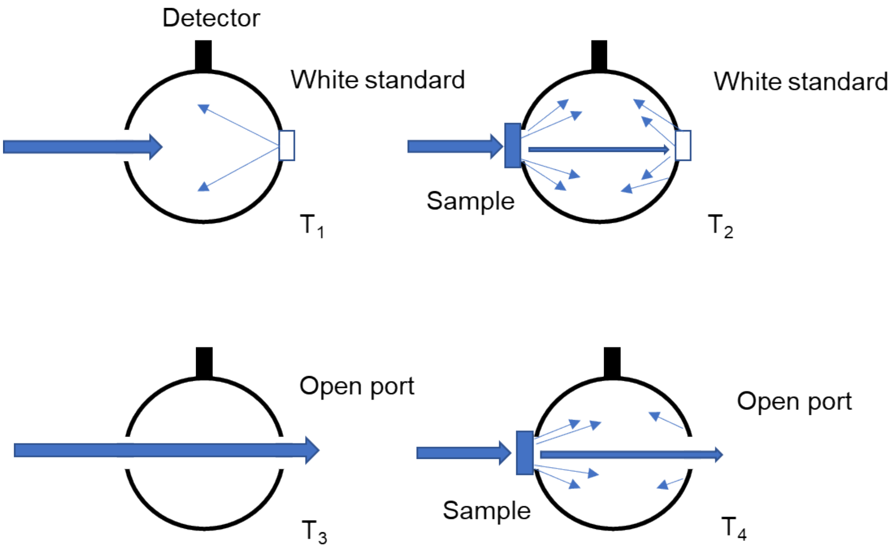

Figure S1. Schematic of the experimental setup for haze measurements. T_1 is the total light transmitted without a sample, T_2 is the total light transmitted with a sample, T_3 is the light scattered by the instrument and T_4 is the light scattered by the instrument and sample. Haze was calculated according to the following equation: $Haze\ (\%) = [T_4/T_2 - T_3/T_1] \times 100\%$.

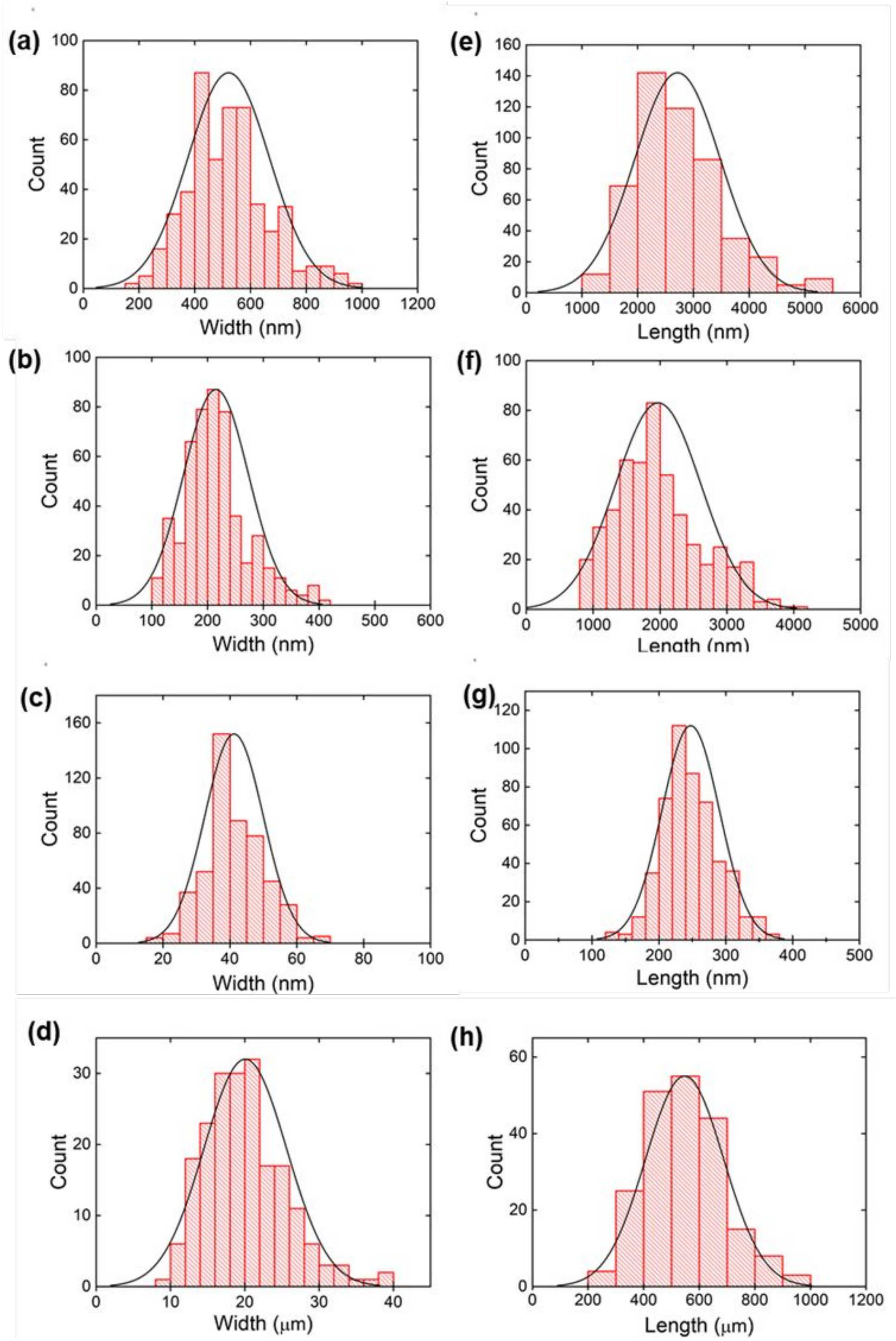

Figure S2. Histograms and fitted log-normal distribution curves for (a) width of CMPs-L, (b) width of CMPs-M, (c) width of CMPs-S, (d) width of CMPs-XL, and (e) length of CMPs-L, (f) length of CMPs-M, (g) length of CMPs-S, (h) length of CMPs-XL. (All the width and length data are obtained from STEM measurements)

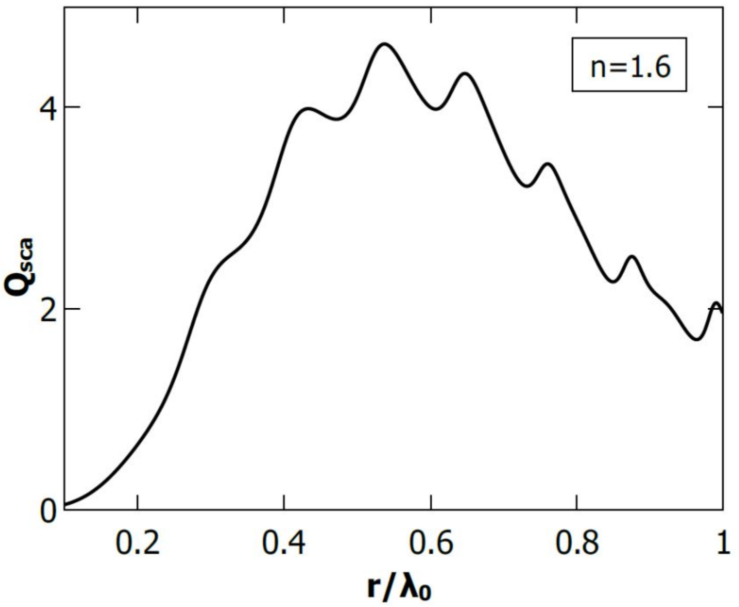

Figure S3. The scattering strength of a Mie-Sphere plotted against the size ratio (radius/wavelength) according to Mie theory.[1]

In Figure S3, the normalized scattering strength of a sphere for a “low refractive index” material was plotted. It can be seen that for increasing size the scattering increases non-monothonic and reaches a maximum before it decreases again drastically. CMPs are optimized to lie in the range of this maximum. This curve explains also why CMPs-XL again scatter less than the CMPs-L but still perform some scattering, leading to haze. Note that the resonant behaviour observed in Figure S3 is not relevant for CMPs as they are non-spherically shaped and have a finite size distribution.

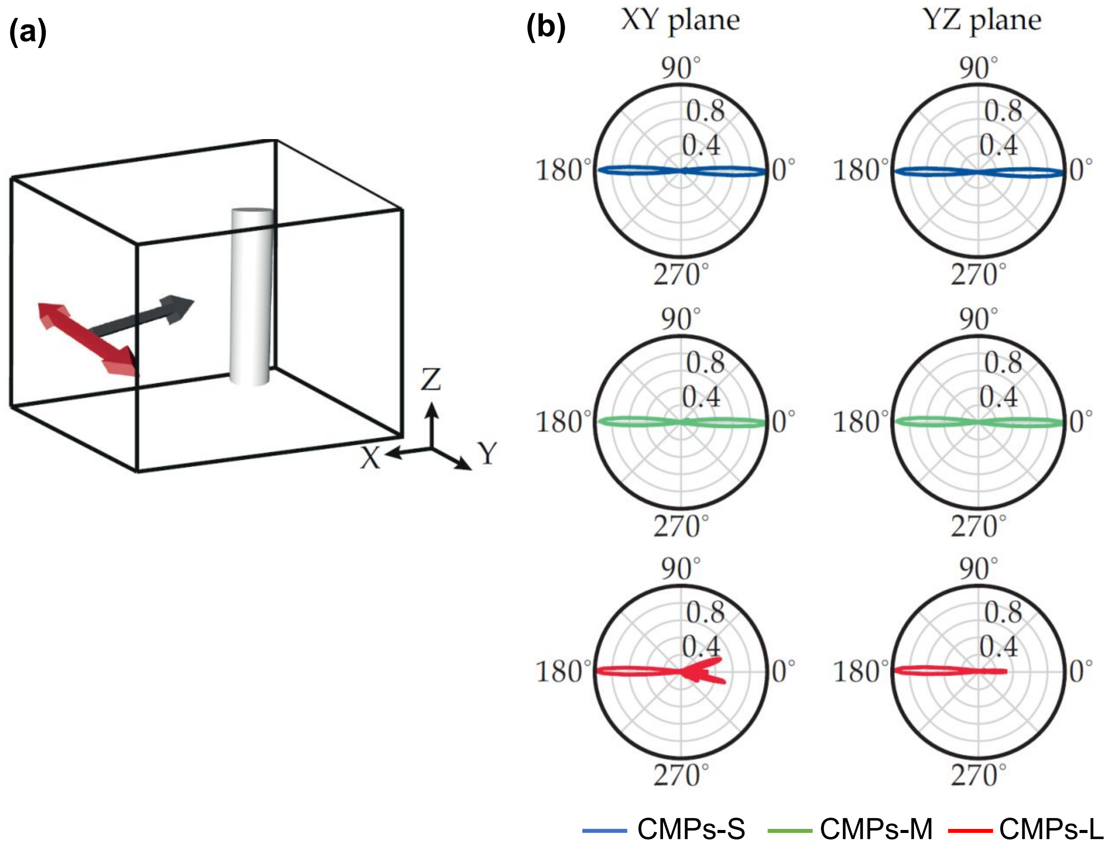

Figure S4. Numerical, three-dimensional, simulations of the single-particle properties of CMPs. (a) Schematics of the simulation setup used to calculate the scattering from CMPs, which were approximated as cylinders with values of diameter and length from Table 1. The incoming light and its polarization are represented with black and red arrows, respectively. The scattered intensity was acquired in all directions. (b) Angular distribution of the scattered light (differential scattering cross section) for the three different types of CMPs. The results presented in (b) were obtained averaging over two orthogonal polarizations. (Numerical FDTD simulations of the optical response of the generated structures were performed in Lumerical).

(a)

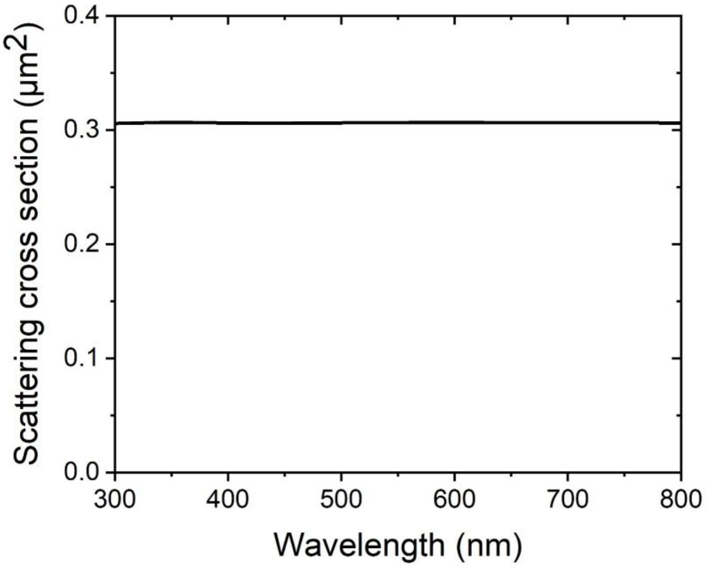

(b)

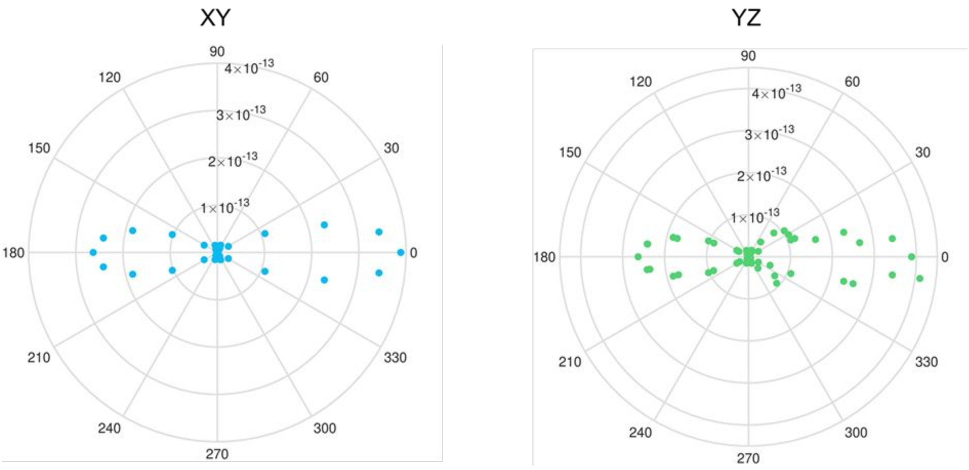

Figure S5. (a) Optical simulations of scattering cross-section of traditional CNCs. (b) Angular distribution of the scattered light (differential scattering cross section) for traditional CNCs. (Numerical simulations of the optical response of the generated structures were performed in Lumerical. The dimensions of CNCs used in simulations are 200 nm for length and 5 nm for diameter).

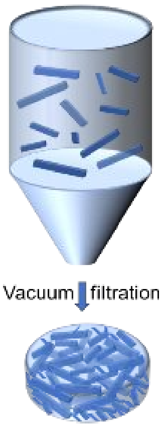

Figure S6. Schematic of the vacuum process of fabrication of free-standing films from cellulose particles. The suspension of CMPs (S, M, L) is filtered through a membrane, when the water is removed, CMPs will form a percolated network. The thickness and/or the filling fraction of CMPs can be adjusted by the filtration time (or the amount of water is removed).

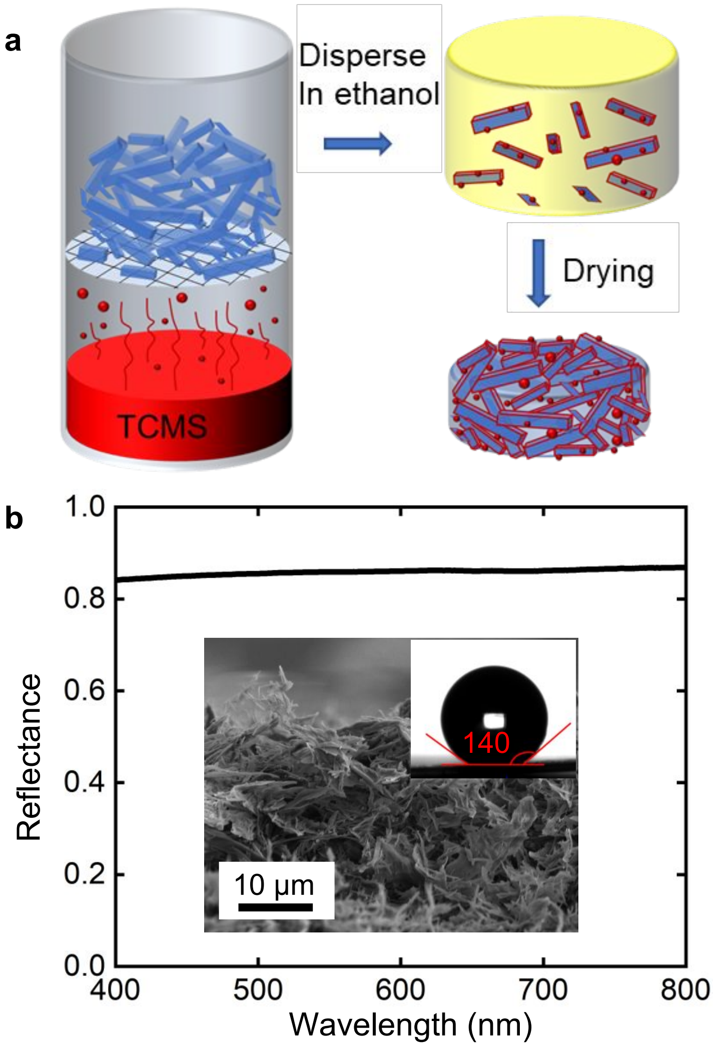

Figure S7. Schematic of the process for hydrophobic treatment of CMPs-L, and the formation

of films which were directly dried in the air, (b) reflectance of the hydrophobic CMPs-L films, measured with an integrating sphere; inset images show the SEM image of the cross-section of the hydrophobic CMPs-L films, and contact angle profile of water droplet on the surface of hydrophobic CMPs-L film.

As shown in Figure S7, a second simplified and scalable method to produce highly scattering films that allows removing the filtration, freezing and freeze-drying steps, has also been developed. The produced CMPs-L were first made partially hydrophobic by trichloromethylsilane (TCMS) vapor treatment and then dispersed in ethanol. Films were then obtained by simply drop-casting the CMPs-L ethanol suspension in the air at room temperature. When treated with TCMS, part of the hydroxyl groups on the surface of CMPs are replaced by -O-$SiCH_3$ moieties,[2] so the hydrogen bonding between hydroxyl groups is significantly reduced during the drying process. Due to the reduced hydrogen bonding and the lower surface tension of ethanol with respect to water, a CMPs-L porous network can be achieved in a thin film without collapsing under the capillary pressure produced by the solvent evaporation.[3]

For the hydrophobic CMPs-L films with a thickness of 25 μm, we observed the reflectivity ranging from 84% at 400 nm to 88% at 800 nm (Figure S7b). Moreover, these highly scattering films composed of hydrophobic CMPs-L show a good hydrophobic response. As shown in Figure S7b (inset image), the water droplet contact angle of these films reaches 140º, close to what is achieved with a superhydrophobic surface,[4] widening their potential for application as an antifouling and self-clean white coating.[5]

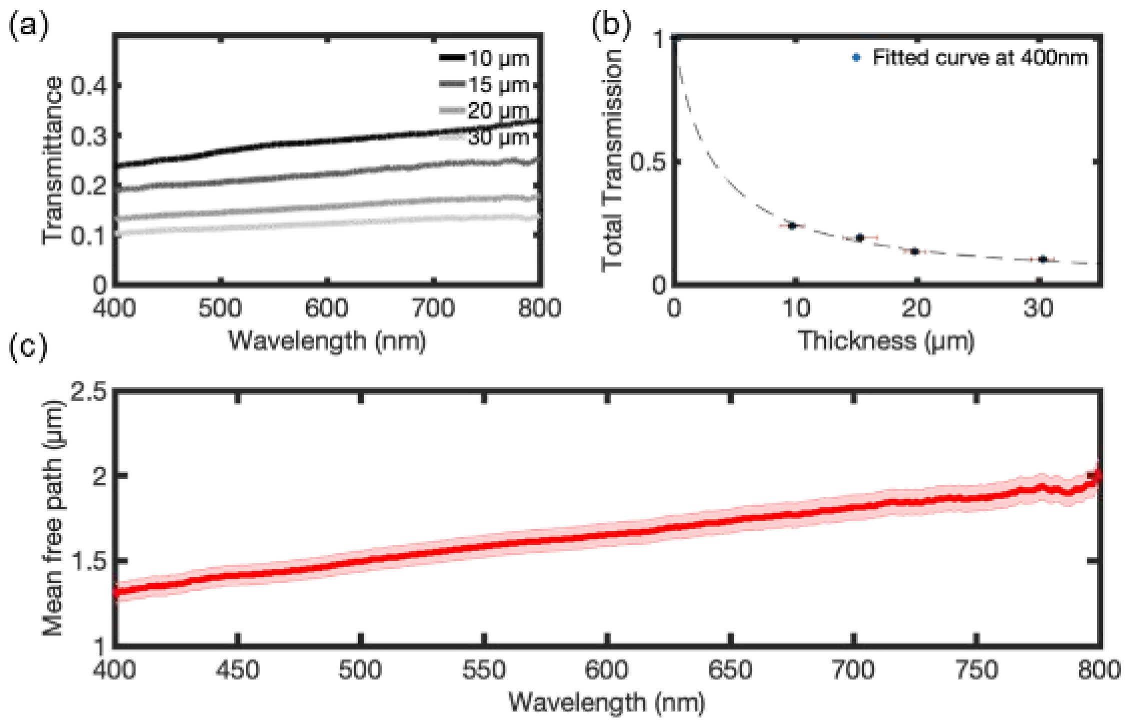

Figure S8. (a) Total transmission data for CMPs-L samples with ff=0.4 and different thickness, measured with an integrating sphere. (b) the fit of the total transmission at a wavelength of 400 nm, (c) the spectral dependency of the transport mean free path.

As shown in Figure S8a and b, measuring samples with different thicknesses allows to precisely estimate the value of the mean free path. This procedure was performed for CMPs-L samples with ff=0.4, the rest of the data in Table 2 are estimate values obtained from the above-mentioned equation and single values of thickness and total transmission. Figure S8c shows the wavelength dependency of the transport mean free path (l_t). The transport was almost wavelength-independent, resembling the shape of the total transmission spectra.

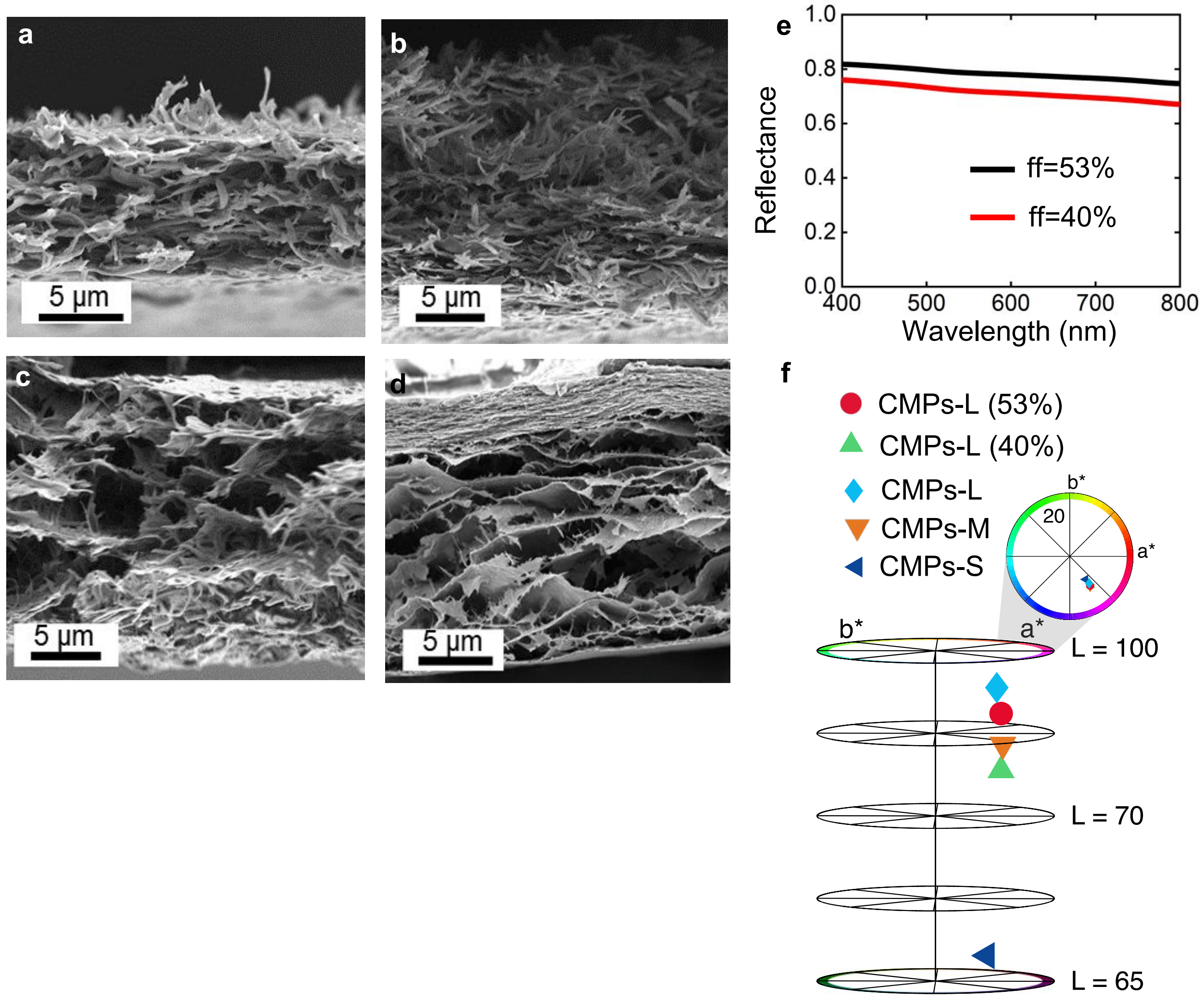

Figure S9. Dependence of the scattering properties on the filling fraction: (a) SEM image of the cross-section of white films made of CMPs-L with ff=53%. Dependence of the scattering properties on the size of the building blocks: SEM images of the cross-section of white films from (b) CMPs-L, (c) CMPs-M, (d) CMPs-S at the same thickness of 25 µm. The films prepared from CMPs-S, which have the smallest size (about 40 nm in width and 228 nm in length) among the four types of CMPs. CMPs-S are prone to pack more densely than CMPs-M and CMPs-L, and the vacuum force applied on the bottom of the wet film is stronger than the surface of the film, creating the gradient in filling fraction. (e) reflectance of white films made of CMPs-L with the thickness of 9 µm at ff=53% and 40%, measured with an integrating sphere. Whiteness comparison:(f) polar plot showing the CIELAB color space coordinates of the spectra in panel.

Morphological characterization of CMP films

When CMPs are exploited to produce porous scattering media, it is also important to investigate of the porous structure during its formation and its final morphology. This is particularly critical

for smaller size CMPs, where the morphology of films is strongly affected by the dynamic of ice formation during freeze-drying. CMPs-M films display more layered and dense structures by packing a few CMPs-M together, see Figure S7c. This effect is even more obvious in CMPs-S films, where clear layered and dense structures are visible near the film surface (Figure S9d). We speculate that ice crystals formed during the freeze-drying push the CMPs at the domain boundaries between different ice crystals, leading to the formation of layered sheets of CMPs-S.[6] In CMPs-L films, the absence of flakes and layers (Figure 2b, S9a, and S9b) is instead prevented probably due to their larger size.

Spectral scattering properties of CMP films

In Figure S9f, we compare the scattering response for the different CMPs-based films in terms of spectral dependence. Whiteness, as discussed in detail in the Supplementary Information, is optimized by increasing the amount of light reflected at different wavelengths and it can be calculated by converting the reflectance spectra in La*b* color-space coordinates.[7] Interestingly, films with CMPs of different sizes have a similar color saturation, i.e., the radial distance from the center of coordinates (a*, b*), but different luminosity, i.e., vertical coordinate (L). Importantly, the reported value of whiteness exceeds one of the CNFs-based systems.[8]

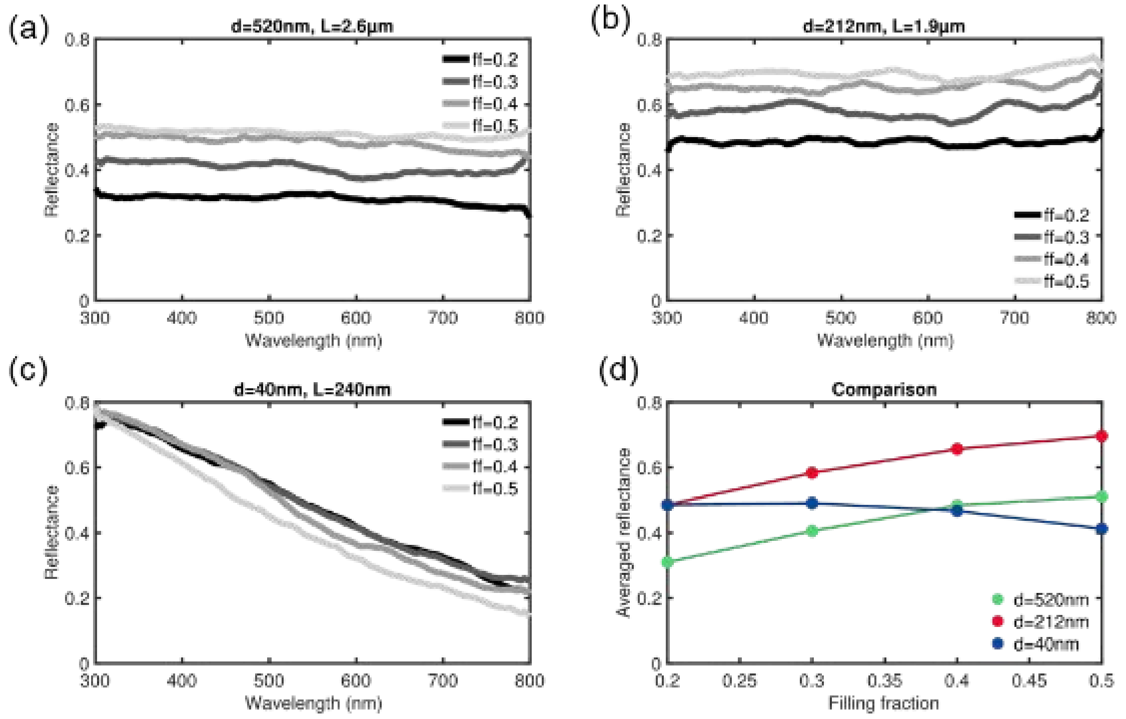

Figure S10. (a-b-c) Simulated optical response for the ensemble of CMPs-L, M, S with different filling fractions. (d) comparison of the data reported in (a-b-c) in terms of their average reflectance over the visible. All simulated systems have a thickness of 10 μm.

Figure S10 shows the dependence on the filling fraction of the scattering properties of CMPs-based systems. As shown in Figure S8a, and experimentally confirmed in Figure S9e, for materials made of CMPs-L particles, increasing the filling fraction leads to an increase of reflectance.

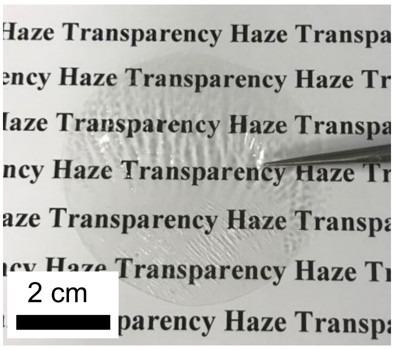

Figure S11. Pure CMC film, upside part of this film is about 1 cm above from the background.

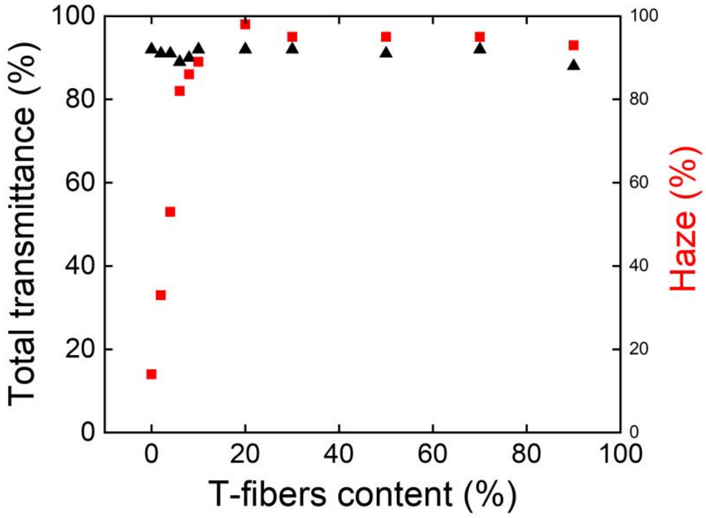

Figure S12. Total light transmittance and haze values of films with different content of CMPs-XL. (For pure CMC films, their transmittance is about 92% and haze is about 14%). All data were measured with an integrating sphere.

Table S1. Transmittance and haze values of various transparent cellulose films at 400-800 nm. (*data was only provided at 550 nm in the original paper)

Films	Materials	Transmittance (T)	Haze (H)	Ref.
Transparent paper	Regenerative cellulose fibers by dissolving-debonding strategy	90-92%	41%*	9
Nanopaper	TEMPO-oxidized pulp after being disintegrated by microfluidizer	90-93%	48-55%	10
Novel nanostructured paper	TEMPO-oxidized wood fiber	93-96%	56-62%	11
Anisotropic transparent paper	Mechanical pressed lignin removed wood slices	89-90%	65-88%	12
Transparent hybrid paper	Wood fibers and nanofibrillated cellulose mixture by filtration	90-92%	70-74%	13
Cellulose composite film	CMC solution infiltrated into common paper	90-91%	80-84%	14
Hazy transparent nanopaper	Cellulose pulps fibrillated by a water-jet nanofibrillation	89-92%	27-87%	15
Super hazy paper	Hot-press of ionic liquid partially dissolved paper	89- 91%	80-91%	16
Our films	CMC and TEMPO oxidized cellulose fiber	89-92%	96-98%	

References

(1) Bohren, C. F.; Huffman, D. R. Absorption and Scattering by a Sphere. In *Absorption and Scattering of Light by Small Particles*; John Wiley & Sons, Ltd: Weinheim, 1998; pp 82–129. https://doi.org/10.1002/9783527618156.ch4.

(2) Cunha, A. G.; Freire, C.; Silvestre, A.; Neto, C. P.; Gandini, A.; Belgacem, M. N.; Chaussy, D.; Beneventi, D. Preparation of Highly Hydrophobic and Lipophobic Cellulose Fibers by a Straightforward Gas–Solid Reaction. *J. Colloid Interface Sci.* **2010**, *344* (2), 588–595. https://doi.org/https://doi.org/10.1016/j.jcis.2009.12.057.

(3) Tejado, A.; Chen, W. C.; Alam, M. N.; van de Ven, T. G. M. Superhydrophobic Foam-like Cellulose Made of Hydrophobized Cellulose Fibres. *Cellulose* **2014**, *21* (3), 1735–1743. https://doi.org/10.1007/s10570-014-0247-x.

(4) Ma, M.; Hill, R. M. Superhydrophobic Surfaces. *Curr. Opin. Colloid Interface Sci.* **2006**, *11* (4), 193–202. https://doi.org/https://doi.org/10.1016/j.cocis.2006.06.002.

(5) Parkin, I. P.; Palgrave, R. G. Self-Cleaning Coatings. *J. Mater. Chem.* **2005**, *15* (17), 1689–1695. https://doi.org/10.1039/B412803F.

(6) Erlandsson, J.; Pettersson, T.; Ingverud, T.; Granberg, H.; Larsson, P. A.; Malkoch, M.; Wågberg, L. On the Mechanism behind Freezing-Induced Chemical Crosslinking in Ice-Templated Cellulose Nanofibril Aerogels. *J. Mater. Chem. A* **2018**, *6* (40), 19371–19380. https://doi.org/10.1039/C8TA06319B.

(7) Jacucci, G.; Bertolotti, J.; Vignolini, S. Role of Anisotropy and Refractive Index in Scattering and Whiteness Optimization. *Adv. Opt. Mater.* **2019**, *7* (23), 1900980. https://doi.org/10.1002/adom.201900980.

(8) Toivonen, M. S.; Onelli, O. D.; Jacucci, G.; Lovikka, V.; Rojas, O. J.; Ikkala, O.;

Vignolini, S. Anomalous-Diffusion-Assisted Brightness in White Cellulose Nanofibril Membranes. *Adv. Mater.* **2018**, *30* (16), 1704050. https://doi.org/10.1002/adma.201704050.

(9) Chen, J.; Han, X.; Fang, Z.; Cheng, F.; Zhao, B.; Lu, P.; Li, J.; Dai, J.; Lacey, S.; Elspas, R.; Jiang, Y.; Liu, D.; Hu, L. Rapid Dissolving-Debonding Strategy for Optically Transparent Paper Production. *Sci. Rep.* **2015**, *5* (1), 17703. https://doi.org/10.1038/srep17703.

(10) Zhu, H.; Xiao, Z.; Liu, D.; Li, Y.; Weadock, N. J.; Fang, Z.; Huang, J.; Hu, L. Biodegradable Transparent Substrates for Flexible Organic-Light-Emitting Diodes. *Energy Environ. Sci.* **2013**, *6* (7), 2105–2111. https://doi.org/10.1039/C3EE40492G.

(11) Fang, Z.; Zhu, H.; Yuan, Y.; Ha, D.; Zhu, S.; Preston, C.; Chen, Q.; Li, Y.; Han, X.; Lee, S.; Chen, G.; Li, T.; Munday, J.; Huang, J.; Hu, L. Novel Nanostructured Paper with Ultrahigh Transparency and Ultrahigh Haze for Solar Cells. *Nano Lett.* **2014**, *14* (2), 765–773. https://doi.org/10.1021/nl404101p.

(12) Zhu, M.; Wang, Y.; Zhu, S.; Xu, L.; Jia, C.; Dai, J.; Song, J.; Yao, Y.; Wang, Y.; Li, Y.; Henderson, D.; Luo, W.; Li, H.; Minus, M. L.; Li, T.; Hu, L. Anisotropic , Transparent Films with Aligned Cellulose Nanofibers. **2017**. https://doi.org/10.1002/adma.201606284.

(13) Fang, Z.; Zhu, H.; Preston, C.; Han, X.; Li, Y.; Lee, S.; Chai, X.; Chen, G.; Hu, L. Highly Transparent and Writable Wood All-Cellulose Hybrid Nanostructured Paper. *J. Mater. Chem. C* **2013**, *1* (39), 6191–6197. https://doi.org/10.1039/C3TC31331J.

(14) Hu, W.; Chen, G.; Liu, Y.; Liu, Y.; Li, B.; Fang, Z. Transparent and Hazy All-Cellulose Composite Films with Superior Mechanical Properties. *ACS Sustain. Chem. & Eng.* **2018**, *6* (5), 6974–6980. https://doi.org/10.1021/acssuschemeng.8b00814.

(15) Hsieh, M.-C.; Koga, H.; Suganuma, K.; Nogi, M. Hazy Transparent Cellulose Nanopaper. *Sci. Rep.* **2017**, *7* (1), 41590. https://doi.org/10.1038/srep41590.

(16) Zhu, H.; Fang, Z.; Wang, Z.; Dai, J.; Yao, Y.; Shen, F.; Preston, C.; Wu, W.; Peng, P.; Jang, N.; Yu, Q.; Yu, Z.; Hu, L. Extreme Light Management in Mesoporous Wood Cellulose Paper for Optoelectronics. *ACS Nano* **2016**, *10* (1), 1369–1377. https://doi.org/10.1021/acsnano.5b06781.
